# Clinical Manifestations and Complications of Children With COVID-19 Compared to Other Respiratory Viral Infections: A Cohort Inpatient Study From Rio de Janeiro, Brazil

**DOI:** 10.3389/fped.2022.934648

**Published:** 2022-07-18

**Authors:** Giuliana Pucarelli-Lebreiro, Marianna Tavares Venceslau, Catherine Crespo Cordeiro, Fernanda Queiroz Maciel, Thiago Dias Anachoreta, Thalita Fernandes de Abreu, Ana Cristina Cisne Frota, Terezinha Marta Pereira Pinto Castiñeiras, Analucia Mendes da Costa, Adriana Cristina da Luz Lopes, Ludmila Nascimento Rodrigues Campos, Luiza Maria Calvano, Maria Angelica Arpon Marandino Guimaraes, Cristina Barroso Hofer

**Affiliations:** ^1^Department of Pediatric Infectious Diseases, Instituto de Puericultura Pediatria Martagão Gesteira, Federal University of Rio de Janeiro, Rio de Janeiro, Brazil; ^2^Department of Preventive Medicine, University Hospital Clementino Fraga Filho, Federal University of Rio de Janeiro, Rio de Janeiro, Brazil; ^3^Department of Pediatrics, Instituto de Puericultura Pediatria Martagão Gesteira, Federal University of Rio de Janeiro, Rio de Janeiro, Brazil

**Keywords:** COVID-19, respiratory viruses infections, children, hospitalized, Brazil

## Abstract

**Introduction:**

The coronavirus disease-2019 (COVID-19) clinical manifestations in children and adolescents are diverse, despite the respiratory condition being the main presentation. Factors such as comorbidities and other respiratory infections may play a role in the initial presentation of severe acute respiratory syndrome coronavirus 2 (SARS-CoV-2) infection. This study aims to describe the epidemiological aspects, clinical, and laboratory manifestations of pediatric patients admitted to a tertiary pediatric hospital in Rio de Janeiro, diagnosed with COVID-19, and compare these with other viral conditions during the first year of the SARS-CoV-2 pandemic.

**Methods:**

All patients under 18 years of age that were admitted with upper airway infection were enrolled and followed up for 30 days. The main dependent variable was the laboratorial diagnosis of SARS-CoV-2, and independent variables were studied through logistic regression.

**Results:**

A total of 533 patients were recruited, and 105 had confirmed SARS-CoV-2 infection. Detection of other viruses occurred in 34% of 264 tested participants. Six patients died (two in SARS-CoV-2 infected group). The variables independently associated with COVID-19 were older age (OR = 1.1, 95% CI = 1.0–1.1), lower leukocytes count at entry (OR = 0.9, 95% CI = 0.8–0.9), and contact with suspected case (OR = 1.6, 95% CI = 1.0–2.6). Patients with COVID-19 presented higher odds to be admitted in an intensive care unit (OR = 1.99, 95% CI = 1.08–3.66).

**Conclusions:**

Even during the SARS-CoV-2 pandemic, several other respiratory viruses were present in admitted pediatric patients. Variables associated with COVID-19 infection were older age, lower leukocytes count at entry, and a domiciliary suspect contact. Although patients with COVID-19 were more frequently admitted to ICU, we did not observe higher mortality in this group.

## Introduction

At the end of December 2019, the first cases of coronavirus disease-2019 (COVID-19), a new disease caused by severe acute respiratory syndrome coronavirus 2 (SARS-CoV-2), were described in Wuhan, China. There was a rapid spread of cases and in March 2020 the World Health Organization (WHO) declared the SARS-CoV-2 pandemic. The first case in Brazil was reported in March 2020 and in February 2022 the country ranked third in the world ranking of number of cases (1).

The disease can affect all age groups, and the pediatric population accounts for 8.1% of the cases reported worldwide (2, 3). However, due to the milder nature of the disease in this population, fewer tests for etiological confirmation are performed, with consequent underreporting, and underestimating the total number of cases (3). Typically, SARS-CoV-2 infection among children and adolescents causes asymptomatic and mild conditions, accounting for only 0.2% of global deaths (3, 4). In Brazil, the lethality rate among children and adolescents hospitalized due to SARS triggered by COVID-19 was 7% (5).

The clinical manifestations in children and adolescents are diverse, despite the respiratory condition being the main presentation. These patients often present non-specific signs and symptoms, very similar to other common viral conditions, making the diagnosis of COVID-19 a challenge in pediatrics (6, 7). In addition, 2-4% evolve more severely, requiring hospitalization and intensive care (8–10). The risk factors for unfavorable evolution as well as the clinical manifestations and evolution of COVID-19 in children with comorbidities are still unknown.

This study aims to describe the epidemiological aspects, clinical and laboratory manifestations, and the outcome of pediatric patients admitted to a tertiary pediatric hospital in Rio de Janeiro, Brazil, diagnosed with COVID-19, and compare these with other viral conditions during the first year of the SARS-CoV-2 pandemic.

## Methods

### Study Design and Participants

This is a prospective cohort study involving children and adolescents hospitalized at the tertiary pediatric hospital of the Federal University of Rio de Janeiro, Instituto de Puericultura e Pediatria Martagão Gesteira (IPPMG), which is a reference center for rheumatology, immunology infectious diseases, and onco-hematology care in Rio de Janeiro, Brazil. The children and adolescents who had suspected COVID-19 diagnosis from 14 April 2020 to 30 April 2021 were enrolled.

All patients under 18 years of age admitted at our institution with upper airway infection (with or without fever), SARS, pneumonia, bronchiolitis, gastroenteritis (diarrhea, nausea, or vomiting), or fever of unknown origin were eligible for inclusion. Exclusion criteria were, older than 18 years old at the time of admission, refusal to participate by parents, or lack of real-time PCR (RT-PCR) testing for SARS-CoV-2.

Children and adolescents were admitted according to medical criteria, and RT-PCR testing for SARS-CoV-2 was performed preferably between the third and fifth days of disease.

### Data Collection

Following enrollment, all participants answered an initial questionnaire and were followed up during hospitalization for the data collection. Thirty days after the onset of symptoms, patients returned for an outpatient clinic visit, and a SARS-CoV-2 serology test was performed.

Variables collected included age, gender, race, socioeconomic status, previous contact with a suspected or confirmed COVID-19 case, duration of breastfeeding, contact with indoor smoking, previous vaccination with Influenza, underlying medical conditions, hospital length of stay, signs and symptoms, oxygen requirement, admission to critical care, other viral coinfections, and laboratory tests.

### Ethical Aspects

Participants and/or their parents/guardians provided written informed consent before participation. The study was reviewed and approved by the IPPMG Ethics Committee, number 30786020.4.0000.5264.

All clinical specimens were obtained in accordance with ethical guidelines.

### Study Definitions

The suspected cases with respiratory manifestations were classified as defined by the Brazilian Ministry of Health (11): Influenza syndrome (IS): acute respiratory condition, characterized by at least two of the following signs and symptoms: fever (even if only reported not measured), nasal obstruction, chills, sore throat, headache, cough, runny nose, olfactory disturbances, or taste disturbances.

Severe acute respiratory syndrome: IS presenting with dyspnea/respiratory discomfort or persistent pressure or pain in the chest or O_2_ saturation under 95% in room air or cyanosis of the lips or face.

Patients with extrapulmonary presentations were classified as: Acute gastroenteritis and meningoencephalitis, depending on the clinical presentation.

Multisystem inflammatory syndrome in children (MIS-C) suspected cases were classified according to criteria established by the Centers for Disease Control and Prevention - CDC (https://www.cdc.gov/mis-c/hcp/) (12).

Immunological suppressor use was classified as treatment with any of the following drugs: calcineurin inhibitors, antimetabolites, mammalian target of rapamycin (mTOR) inhibitors, corticosteroids, cyclophosphamide, rituximab, alemtuzumab, Interleukin 6 (IL-6) inhibitors, and anti-Tumour Necrosis Factor alpha (TNF-α) inhibitors.

We classified a participant as a case when RT-PCR for SARS-CoV-2 was positive or undetermined for any clinical sample (nasopharyngeal or oropharyngeal swab, bronchoalveolar lavage, stool, or cerebrospinal fluid), if serological results were positive (IgM and IgG or IgG) after 30 days of initial symptoms or if the participant met the criteria for MIS-C. We consider reagent IgG as a confirmatory laboratory criterion only in unvaccinated individuals, without previous laboratory diagnosis for COVID-19 and who presented compatible signs and symptoms in the first 6 months of study (and the first 6 months of the pandemic).

Patients with negative SARS-CoV-2 and serological tests were compared with those with any positive results.

The date of symptom onset was defined as the day when the first symptom or sign occurred.

### Sample Collection and Viral Detection

The specimens collected varied according to the patient's symptoms. Nasopharyngeal and nasal swabs specimens were collected with synthetic fiber swabs; each swab was inserted into a separate sterile tube containing 2 ml of phosphate-buffered saline (PBS). In intubated or tracheostomized patients, tracheal aspirate specimens were obtained the same way. Spinal fluid and stool were collected in sterile specimen containers. Specimens were immediately processed at the Virology Laboratory or stored at 4°C until processing. Nucleic acid extraction was performed manually with the Bio Gene Viral DNA/RNA extraction kit (Bioclin). SARS-CoV-2 RNA was detected using both the CDC 2019-novel coronavirus (nCoV) Real-Time RT-PCR Diagnostic Panel and Bio-Manguinhos SARS-CoV-2 Molecular Kit on the 7500 Real-Time PCR System (Applied Biosystems™). The Charité/Berlin protocol was used, and samples were considered SARS-CoV-2 positive when both E and RdRP target genes were detected, and if the CT value was not higher than 37.

Additional diagnostic tests were performed for children aged 0–3 months (multiplex respiratory PCR panel – FilmArray-Biofire Diagnostics) and those aged 3 months−2 years (Influenza and Respiratory Syncytial virus PCR tests). SARS-CoV-2 serology was performed using a chemiluminescence immunoassay (Kit Maglumi 2019-nCOV IgG IgM). All assays were performed according to manufacturer instructions. Tests were performed according to hospital unit availability for testing.

### Statistical Analyses

Data were collected and a dataset was built using Access software, version 2019, for Windows 10. All variables distribution were studied. Categorical variables were described by frequency and percentage; continuous variables distributions were studied graphically and were described by the median and interquartile range (IQR). Bivariate analysis was performed using Fisher's Exact test (categorical variables) and Mann–Whitney test (continuous variables). Independent variables with *p*-value < 0.2 were included in a multivariate analysis model, using backward stepwise logistic regression. Variables with <5% of frequency were not added to the multivariate analysis model. The main dependent variable was the laboratorial diagnosis of SARS-CoV-2 and pediatric intensive care unit (PICU) admission. Models with and without interactions were tested using the −2 log-likelihood test. The fitness of the model was evaluated using the Hosmer–Lemeshow test. An alternative hypothesis was accepted if the comparison *p*-value was ≤ 0.05. All data were analyzed using the STATA software, version 15, Texas, USA.

## Results

### Diagnosis Confirmation

A total of 533 patients were recruited during the study period, 105 patients had confirmed SARS-CoV-2 infection and 428 had negative results. The diagnosis was confirmed by RT-PCR in 79 patients (15%). The remaining 26 (5%) were confirmed by detection of IgG antibodies about 30 days after clinical infection.

### Demographic and Epidemiological Features

The median age was 44 months: 45 months for the COVID-19 confirmed children and 24 months in the non-COVID19 group (*p* < 0.01), 286 were male (55%), 346 (68%) non-white, 329 (65%) had prior comorbidities, and 31 (15%) were using immunosuppressive drugs at hospital admission. A total of 98% of the children's parents knew about the risk of COVID-19 and declared that preventive measures were adopted. None of these characteristics presented a statistical difference between the COVID-19 and the non-COVID-19 groups. A contact with confirmed or suspected case in the household was reported for 154 patients (29%): 39% in the COVID-19 group and 26% in the non-COVID-19 group (*p* < 0.01; Table 1).

**Table 1:** Demographic and epidemiological data of pediatric patients hospitalized with suspected COVID-19 diagnosis of both groups.

**Characteristic**	**Total *N* = 533 (100%)**	**COVID positive *N* = 105 (20%)**	**Non-COVID *N* = 428 (80%)**	***p*-value**
Age in months, median (range)	44 (1–192)	45	24	<0.01
Sex				
Male	286 (55%)	57 (54%)	229 (53%)	0.73
Female	247 (45%)	48 (46%)	199 (47%)	
Race				
White	187 (35%)	37/101 (36%)	124/406 (30%)	0.68
Non-white	346 (65%)	64/101 (64%)	282/406 (70%)	
Family income per person (in minimum wages), median (range)	0.3 (0–4)	0.3 (0–4)	0.3 (0.03)	0.64
Breastfeeding	280 (54%)	62/101 (61%)	218/420 (51%)	0.09
In-house smoking	136 (27%)	24/95 (25%)	112/396 (28%)	0.61
Number of rooms per household, median (range)	5 (2–11)	5.0 (2–11)	5.0 (1–11)	0.31
Living in a house with running water and sewage	492 (94%)	93/99 (94%)	399/419 (95%)	0.60
Household/family contact with a suspected/confirmed COVID-19 case	206 (39%)	41/105 (39%)	113/428 (26%)	0.01
Prior Influenza vaccination	294 (71%)	53/82 (65%)	241/333 (72%)	0.13
Weight (in kilograms), median (range)	12 (3–92)	13 (3–71)	12 (3–92)	0.58
Chronic disease	329 (65%)	67/98 (68%)	263/403 (65%)	0.55
Use of immunosuppressive medication	31 (15%)	22/164 (13%)	9/50 (2%)	0.49

### Clinical Presentation and Laboratorial Findings

At admission, the most common symptoms were cough (71%), fever (66%), tachypnea (60%), and dyspnea (53%), there were no differences in initial symptoms between the COVID-19 and the non-COVID-19 groups. Furthermore, a low blood oxygen saturation level (SpO_2_ <95%) was observed in 37% of cases. The main clinical presentation was SARS (53%), respiratory tract infection (19%), gastroenteritis (12%), meningoencephalitis (2%), MIS-C (2%), and others (12%). Among those with diarrhea, 75% presented fever, 62% cough, and 37% dyspnea.

The median of hemoglobin, white blood cells, and platelets was normal. C-reactive protein (CRP) ranged from negative (below the detection limit) to 846 mg/dl (median 45). The median of white blood cell count was lower in patients with SARS-CoV-2 (10.364 vs. 12.207 cells/mm^3^, *p* = 0.04), and this group had shorter hospital stay (84 vs. 352 days, *p* = 0.04; Table 2).

**Table 2:** Clinical features, laboratory findings and diagnosis confirmation of pediatric patients hospitalized with suspected COVID 19 diagnosis.

**Characteristic on hospital admission**	**Total *N* = 533 (100%)**	**COVID positive *N* = 105 (20%)**	**COVID negative *N* = 428 (80%)**	***p*-value**
Fever	349 (66%)	72/105 (69%)	278/424(65%)	0.64
Cough	376 (71%)	68/104 (65%)	308/425 (72%)	0.31
Sore throat	54 (10%)	12/102 (12%)	42/414 (10%)	0.49
Runny nose	267 (51%)	45/102 (44%)	222/417 (53%)	0.12
Anosmia	4 (0.8%)	1/101 (0.1%)	3/419 (0.7%)	0.45
Ageusia	3 (0.6)	0/102 (0%)	3/420 (0.7%)	0.29
Tachypnea	312 (60%)	53/103 (51%)	259/416 (62%)	0.07
Dyspnea	274 (53%)	48/100(48%)	226/415 (54%)	0.265
Pulse oxygen saturation under 95%	182 (37%)	36/96 (36%)	146/398 (37%)	0.90
Hypotension	11 (2%)	2/84 (2%)	9/349 (3%)	1.00
Worsening of underlying disease	173 (35%)	34/90 (38%)	139/382 (37%)	0.80
Rash	50 (10%)	6/100 (6%)	44/413 (11%)	0.19
Abdominal pain	127 (25%)	32/87 (37%)	95/338 (28%)	0.11
Diarrhea	137 (27%)	28/101 (28%)	109/413 (26%)	0.80
Vomiting	319 (62%)	42/102 (41%)	156/415 (37%)	0.57
Hemoglobin (g/dl), median (range)	11 (2–18)	10.7 (2–18)	11.1 (3–16)	0.10
Leukocytes (cells/mm^3^), median (range)	10.405 (1–73.230)	8.565 (1–60.610)	10.800 (5–73.230)	0.04
Platelets (cells/mm^3^), median (range)	329.000 (4,000–977.000)	313.000 (5,000–977.000)	335.000 (4.000–728.000)	0.12
C-reactive protein – CRP (mg/dl), median (range)	17 (0–846)	46 (0–406)	45 (0–846)	0.94
Erythrocyte sedimentation rate-ESR, median (range)	16 (0–493)	19 (2–120)	15 (0–493)	0.48
Length of hospital stay (days), median (range)	5 (1–733)	6 (1–120)	5 (1–733)	0.12
Admission in Intensive care unit	85 (16%)	23/105 (22%)	62/428(14.5%)	0.07
Deaths	6 (1%)	2/105 (1.9%)	4/428 (0.9%)	0.33
Initial presentation with Flu^*^-like syndrome	80 (15%)	19/87 (21%)	61/336 (18%)	0.44
Initial presentation with SARS^**^	223 (53%)	44/87 (50%)	179/336 (53%)	0.71
Initial presentation with MIS-C^***^	8 (2%)	2/87 (3%)	6/336 (2%)	0.67
Initial presentation with Acute gastroenteritis	51 (12%)	11/87 (12%)	40/336 (12%)	0.85
Initial presentation with Meningoencephalitis	8 (2%)	3/87 (3.5%)	5/336 (1.5%)	0.21

* *Flu, influenza*;

** *SARS, severe acute respiratory syndrome*;

*** *MIS-C, Multisystem inflammatory syndrome in children*.

### Coinfection

Detection of other viruses occurred in 34% of 264 tested participants. Coinfection was observed in 15 (6%) of the 264 tested, with 9 (3.5%) coinfection between SARS-CoV-2 and other viruses. Also, other respiratory viruses were detected in 81 patients in the non-COVID-19 group: in 75 patients just other one virus was detected, while in six patients more than one was detected. The multiplex respiratory panel was performed on 42 patients, 128 were screened for influenza, and 94 for respiratory syncitial virus (RSV) by RT-PCR. RSV was isolated in 70/136 patients tested (51%); Rhino/enterovirus in 11/42 tested (26%); influenza in 6/128 tested (5%), parainfluenza in 2/42 tested (4%), and adenovirus in 1/42 (2%). We did not detect any bacterial or fungal disease in this study population (Table 3).

**Table 3:** Coinfections at the hospital admission.

**Coinfection**	**Total *N* (%)**
SARS-CoV-2 + RSV^*^	5/136 (4%)
SARS-CoV-2 + influenza	1/128 (1%)
SARS-CoV-2 + rhino/enterovírus	1/42 (3%)
SARS-CoV-2 + rhino/enterovirus + RSV	1/42 (3%)
SARS-CoV-2 + norovirus	1/42 (3%)
Rhino/enterovirus + RSV	5/42 (11%)
Rhino/enterovirus + RSV + adenovírus	1/42 (3%)

* *RSV, respiratory syncytial virus*.

### Multivariate Analysis

In the multivariate analysis, the variables associated with COVID-19 diagnosis were higher mean age, lower mean leukocyte count, and positive history of contact with a suspected or confirmed case (Table 4).

**Table 4:** Variables associated with COVID-19 diagnosis – multivariate analysis.

**Variable**	OR^*^	95% CI^**^	***p*-value**
Age (per month)	1.1	1.0–1.1	<0.01
Leukocytes (cell/mm^3^)	0.9	0.8–0.9	0.06
Contact with suspected case	1.6	1.0–2.6	0.05

* *OR, odds ratio*;

** *CI, confidence interval*.

*Hosmer Lemeshaw test p = 0.39*.

### Outcomes

The median hospital length of stay was 5 days and 98% were discharged without any sequelae. Patients with SARS-CoV-2 had shorter hospital stay (84 vs. 352 days, *p* = 0.04), but admission at the intensive care unit (PICU) was more frequent in this group (22 vs. 14,5%, *p* = 0.07). Six patients (1%) died, median age was 48 months, two in the COVID-19 group and 4 in the non-COVID-19 group (1.9 vs. 0.9%, *p* = 0.33). In COVID-19-group, one patient had a chronic granulomatous disease and the other was previously healthy; in the negative group, the four had previous comorbidities (two with nephropathy, one with acute lymphocytic leukemia, and one with cardiopathy). None of them had coinfection detected.

We also depicted factors possibly associated with PICU admission. Patients in the COVID-19 group presented higher (almost twice) odds to be admitted to the PICU when compared with those from the COVID-19 negative group, even adjusting for respiratory distress and presence of comorbidities (Table 5).

**Table 5:** Epidemiological data, clinical features and diagnosis confirmation of pediatric patients hospitalized with suspected COVID-19, comparing ICU admitted.

**Characteristic**	**Total *N* = 533 (100%)**	**ICU admitted *N* = 85 (16%)**	**ICU not admitted *N* = 448 (84%)**	***p*-value**	Adjusted^*^OR (95% CI)^**^
Age in months, median (range)	44 (1–192)	24 (1–192)	24 (0–192)	0.71	
Sex					
Male	286 (55%)	46 (57%)	240 (55%)	0.44	
Female	230 (45%)	35 (43%)	195 (45%)		
Race					
White	161 (32%)	22 (29%)	139 (32%)	0.59	
Non-white	346 (68%)	55 (71%)	291 (68%)		
Fever at admission	350 (66%)	53 (62%)	297 (67%)	0.45	
Cough	376 (71%)	67(80%)	309(69)	0.04	
Tachypnea	312 (60%)	60 (74%)	252 (58%)	<0.01	
Dyspnea	274 (53%)	59 (71%)	215 (50%)	<0.01	
Pulse oxygen saturation under 95%	182 (37%)	52 (68%)	130 (31%)	<0.01	4.08 (2.36–7.03)
Hypotension	11 (2%)	6 (7.4%)	5 (1.6%)	<0.01	
COVID-19 positive	105 (20%)	23 (27%)	82 (18%)	0.07	1.99 (1.08–3.66)
Weight (in kilograms), median (range)	12 (3–92)	13 (3–71)	12 (3–92)	0.56	
Chronic disease	329 (65%)	62 (78%)	267 (63%)	0.19	2.08 (1.11–3.88)
Use of immunosuppressive medication	31 (15%)	26 (14%)	5 (14%)	0.99	

* *Hosmer Lemeshaw test p = 0.39*.

** OR, odds ratio; **95%CI, 95% confidence interval.

## Discussion

In this study, we were able to describe the clinical, laboratorial, and epidemiological profile of children and adolescents hospitalized with COVID-19 during the first year of the pandemic in Brazil, compared with hospitalizations for possibly other viral conditions, which are frequent in this population.

Most patients who required hospitalization were male and had comorbidities, with higher mean age among patients with COVID-19. To date, there is still some divergence in the literature regarding the age group at greatest risk for COVID-19 infection, and there is a description of a bimodal pattern of severity, with infants under 1 year of age and adolescents being the groups described with the highest risk of developing serious disease (13–16). In the COVID-19 group, we did not observe any difference neither in the need for intensive care nor the mortality related to age.

The frequency of comorbidities in children and adolescents hospitalized due to SARS-CoV-2 was similar to that of patients hospitalized for other viral conditions, and higher when compared to that described in the literature, which hovers around 40% (13, 14, 17). These findings can be explained by the differentiated profile of patients treated at a tertiary hospital and are in agreement with previous studies that suggest that children with comorbidities are at greater risk of hospitalization due to COVID-19 (14, 18).

Approximately half of the patients with a confirmed diagnosis of COVID-19 had a history of contact with a suspected or confirmed case, predominantly in the home environment. This finding reinforces the importance of the epidemiological history at the time of clinical suspicion, especially for differentiation with other diagnostic hypotheses, and corroborates the hypothesis that children acquire the virus mainly through contact with infected adults (19). This finding may be influenced by the fact that public schools were closed due to public safety measures during the study period. Indeed, in a cross-sectional Indonesian study of 1,018 children suspected of COVID-19 disease, factors associated with the 94 confirmed COVID-19 cases were history of a previous travel or contact with an infected person (20). In another Spanish study of children with respiratory symptoms, being older, obesity, and household contact with a case, were associated with a COVID-19 (21).

There was no great difference between the initial clinical manifestations of patients with COVID-19 and other viral conditions, with fever, cough, and tachydyspnea being the most frequent signs and symptoms among patients with a confirmed diagnosis, similar to that described in the literature (13, 15, 22). Despite the predominance of respiratory symptoms and fever in the initial clinical presentation, these were absent in 30% of COVID-19 cases. In addition, approximately a quarter of these patients, presented predominantly gastrointestinal symptoms, similar to what was described in other studies in this population, and symptoms common among adults, such as ageusia and anosmia, were infrequent even among adolescents (12, 18). The variability of clinical presentation imposes a major diagnostic challenge in this age group and reinforces the need for high clinical suspicion of SARS-CoV-2 infection in children with systemic symptoms, even in the absence of fever or respiratory symptoms.

During the study period, the main agent isolated in febrile patients was SARS-CoV-2, followed by RSV and Rhinovirus/Enterovirus. Among patients with COVID-19, coinfection with other viruses commonly seen within the age group was observed in 3% of cases. Our prevalence of SARS-CoV-2 and RSV coinfection with other viral agents was 5% for RSV and 3% for Rhinovirus/enterovirus. In the literature, the prevalence of coinfection varies from 2 to 18% (23, 24). There are few studies in the literature on coinfection in children, and there is no consensus on the interference it has on the clinical course.

Our study has limitations, it was carried out in a tertiary institution, with a different patient profile, which could contribute to greater severity and longer hospital stay. In addition, during the study period, there were variations in relation to the tests performed to investigate other etiological agents, due to the routine and availability of tests in the unit. However, the study was carried out for a long period, covering a large number of admitted children from a single center in a middle-income developing country, and not secondary data analysis, contributing significantly to the current knowledge of the impact of SARS-CoV-2 in children and adolescents. Given the challenges of diagnosing the disease in pediatrics, further studies to evaluate its clinical profile and evolution in the face of new variants and in the vaccination scenario of this group will be necessary.

## Data Availability Statement

The raw data supporting the conclusions of this article will be made available by the authors, without undue reservation.

## Ethics Statement

The studies involving human participants were reviewed and approved by the IPPMG Ethics Committee, Number 30786020.4.0000.5264. Written informed consent to participate in this study was provided by the participants' legal guardian/next of kin.

## Author Contributions

GP-L, MV, TFA, AF, MG, and CH conceptualized and designed the study, drafted the initial manuscript, and reviewed and revised the manuscript. GP-L, CH, CC, FM, TDA, AC, AL, LNRC, and LMC designed the data collection instruments, collected data, carried out the initial analyses, and reviewed and revised the manuscript. TC, TFA, AF, MG, and CH conceptualized and designed the study, coordinated and supervised data collection, and critically reviewed the manuscript for important intellectual content. All authors approved the final manuscript as submitted and agree to be accountable for all aspects of the work.

## Funding

All phases of this study were supported by CNPq – PQ2 for CH and FAPERJ for CH.

## Conflict of Interest

The authors declare that the research was conducted in the absence of any commercial or financial relationships that could be construed as a potential conflict of interest.

## Publisher's Note

All claims expressed in this article are solely those of the authors and do not necessarily represent those of their affiliated organizations, or those of the publisher, the editors and the reviewers. Any product that may be evaluated in this article, or claim that may be made by its manufacturer, is not guaranteed or endorsed by the publisher.
